# Linking stress-driven microstructural evolution in nanocrystalline aluminium with grain boundary doping of oxygen

**DOI:** 10.1038/ncomms11225

**Published:** 2016-04-13

**Authors:** Mo-Rigen He, Saritha K. Samudrala, Gyuseok Kim, Peter J. Felfer, Andrew J. Breen, Julie M. Cairney, Daniel S. Gianola

**Affiliations:** 1 Department of Materials Science and Engineering, University of Pennsylvania, Philadelphia, Pennsylvania 19104, USA; 2 Australian Centre for Microscopy and Microanalysis, School of Aerospace, Mechanical and Mechatronic Engineering, University of Sydney, Sydney, New South Wales 2006, Australia; 3 Department of Materials, University of California Santa Barbara, Santa Barbara, California 93106, USA

## Abstract

The large fraction of material residing at grain boundaries in nanocrystalline metals and alloys is responsible for their ultrahigh strength, but also undesirable microstructural instability under thermal and mechanical loads. However, the underlying mechanism of stress-driven microstructural evolution is still poorly understood and precludes rational alloy design. Here we combine quantitative *in situ* electron microscopy with three-dimensional atom-probe tomography to directly link the mechanics and kinetics of grain boundary migration in nanocrystalline Al films with the excess of O atoms at the boundaries. Site-specific nanoindentation leads to grain growth that is retarded by impurities, and enables quantification of the critical stress for the onset of grain boundary migration. Our results show that a critical excess of impurities is required to stabilize interfaces in nanocrystalline materials against mechanical driving forces, providing new insights to guide control of deformation mechanisms and tailoring of mechanical properties apart from grain size alone.

## Abstract (web summary)

Nanocrystalline metals are ultra-strong because of the large fraction of material at the grain boundaries, but this also leads to instability under applied loads. Here, the authors deepen our understanding of this by linking stress-driven motion of grain boundaries to grain boundary chemistry.

## Graphical abstract

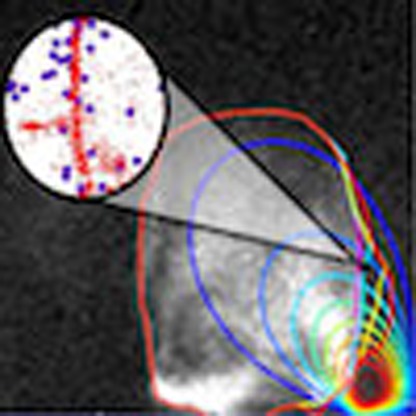

Nanocrystalline (NC) metals and alloys, polycrystals with grain sizes less than ∼100 nm, offer a suite of appealing mechanical properties for structural applications^1^,^2^,^3^,^4^, including high strength and hardness^5^,^6^, enhanced fatigue resistance^7^,^8^ and unprecedented tribological robustness^9^,^10^. These virtues derive from the large fraction of materials that reside at grain boundaries (GBs). At the same time, this high fraction of interfacial materials is responsible for deleterious effects such as thermal instability^11^,^12^ and relatively poor damage tolerance^13^,^14^ in nominally pure NC metals. Despite recent efforts^15^,^16^,^17^,^18^ to synthesize thermally stable NC metals via segregation of alloying species to the GBs, many of these materials, when subjected to mechanical loads, still exhibit grain growth^8^,^10^,^19^,^20^ that can lead to marked changes in mechanical response^21^,^22^,^23^. However, the relationship between thermal and mechanical stability is still not obvious. Many present and future applications of NC metals and alloys such as robust coatings, electrical interconnects, micro- and nano-electro-mechanical systems, and soft magnets involve extreme mechanical duress that can activate microstructural evolution and in turn dynamically change material properties during the lifetime of the device. Characterization and control of mechanical stability in these materials will therefore play an essential role in prediction and optimization of their performances.

While the occurrence of stress-driven grain growth can lead to decreases in strength (via Hall–Petch scaling), it has been shown to markedly enhance tensile ductility via crack-tip blunting^24^. Atomistic simulations have pointed to a critical shear stress needed to promote coupled GB migration^25^,^26^, speculated to be the mechanism underlying stress-driven grain growth as experimentally observed^27^,^28^. Thus, if this threshold can be raised above the macroscopic yield stress, microstructural evolution should only occur in the vicinity of stress concentrators such as crack tips. While manipulation of GB chemistry has been shown in atomistic simulations to stabilize the NC microstructure and control the threshold for stress-driven GB migration^29^,^30^, no experiments have yet quantitatively determined this critical stress, let alone its dependence on the GB excess of doping solutes. Indeed, mapping of the character dependence of GB migration in realistic NC ensembles subjected to far-field stress and linking post-mortem observations of grain growth to the incipient GB migration has yielded limited success. Nevertheless, the successful characterization and control of the GB dopant concentration and thus the thermal and mechanical stability could lead to a new paradigm of alloy design, wherein the critical stress for GB migration could be tailored to impart an unprecedented combination of high strength (from a stable microstructure under ordinary loads) and triggered damage tolerance (from a microstructure that may evolve under stresses concentrated at potential failure sites).

Here we employ quantitative *in situ* nanoindentation inside a transmission electron microscope (TEM) to impart localized mechanical stresses on targeted individual GBs within NC Al films and demonstrate a positive correlation between the critical shear stress required for the onset of GB migration and the GB excess of clustered O atoms, as determined from three-dimensional atom-probe tomography (APT). Measurements of a variety of film compositions and distinct GBs uncover the crossover of the governing deformation mechanisms between dislocation motion and stress-driven GB migration, both directly observed in TEM, thereby providing an estimate for the critical GB excess of impurities necessary for retaining mechanical stability of the NC microstructures.

## Results

### Grain growth induced by *ex situ* nanoindentation

**Figure 1:**
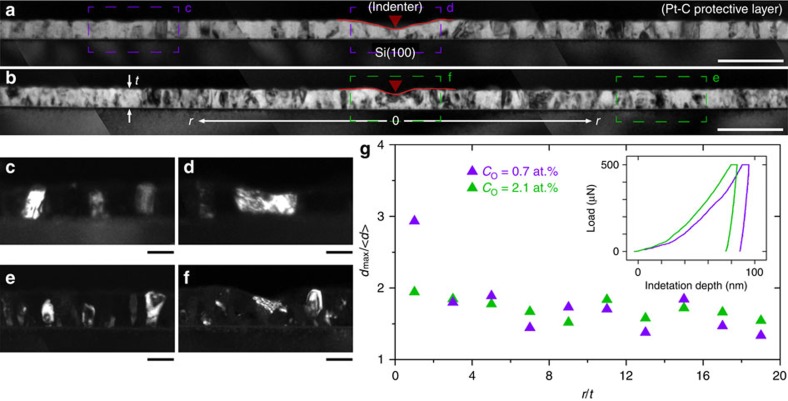
Post-mortem observation of grain growth induced by nanoindentation and its suppression by impurities. (**a**,**b**) Bright-field TEM images of cross-sectional specimens extracted across indents in the NC Al films with global O content (*C*_O_) of (**a**) 0.7 and (**b**) 2.1 at.%. Red lines show surface profiles of indents. Scale bars, 500 nm. (**c**–**f**) Dark-field TEM images of individual grains (**c**,**e**) far from and (**d**,**f**) just beneath indents show occurrence and suppression of grain growth in the film with (**c**,**d**) low and (**e**,**f**) high *C*_O_, respectively. The images correspond to the outlined boxes in (**a**,**b**). Scale bars, 100 nm. (**g**) Quantification of mechanical grain growth. The maximum grain size (*d*_max_) in each lateral bin normalized by averaged grain size of the film (〈*d*〉) is plotted versus the distance to indent (*r*) normalized by film thickness (*t*). Bin size is 2*t*. Inset: indentation load-depth curves of the two films.

NC Al films with thicknesses (*t*) around 160 nm were deposited on Si(100) substrates and micro-machined Si wedges^31^,^32^. The global content of O impurities (*C*_O_) in these films varied from 0.7 to 2.1 at.%, as controlled by confocal co-sputtering of pure Al and α-Al_2_O_3_ targets. More details of film deposition and characterization are described in Supplementary Figs 1–5 and our previous work^33^. The microstructural instability of these films under mechanical stresses was first investigated by instrumented nanoindentation on the films deposited on Si(100) substrates. Cross-sectional specimens across indents were then lifted out inside a focused ion beam/scanning electron microscope (FIB/SEM) from the films with *C*_O_ of 0.7 and 2.1 at.%, as shown in Fig. 1a,b, respectively. In the regions far from indents, which resembled the as-deposited microstructure, both films showed a predominance of columnar grains with the major axis aligned out of plane (Fig. 1c,e). In contrast, the grains beneath the indent showed extraordinary in-plane expansion in the purest film (Fig. 1d), whereas such grain growth was suppressed in the most impure film (Fig. 1f). For further quantification of the grain size evolution in post-deformed regions, each cross-sectional specimen was laterally divided into separate regions using a bin size of 2*t*. The maximum grain size measured in each bin (normalized by the average grain size of the film) was used as a metric for discontinuous grain growth^28^ and plotted versus the distance to the indent (normalized by *t*). As Fig. 1g shows, grain growth was driven by the higher stress near the indent and suppressed due to the increase of *C*_O_, indicating a higher critical stress required for microstructural evolution in more impure films in view of equivalent maximum loads (and thus similar intensity of the stress field) applied to the two films (see inset of Fig. 1g). More details of this quantification are provided in Supplementary Figs 6 and 7. These results are consistent with observations that showed suppression of stress-driven grain growth by O impurities near the fracture ends of NC Al films following micro-tensile tests^21^,^33^.

### GB-targeted, quantitative *in situ* TEM nanoindentation

**Figure 2:**
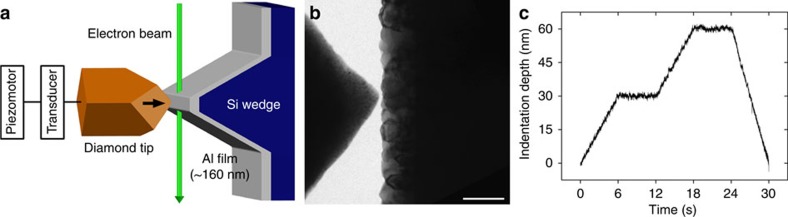
Layout of quantitative *in situ* TEM indentation tests. (**a**) Schematic of experimental set-up showing thin film deposited on a wedge-shaped substrate to enable electron transparency at the wedge apex, as well as provisions for instrumented measurements and alignment. (**b**) Bright-filed TEM image of diamond tip and NC Al film on Si wedge substrate. Scale bar, 200 nm. (**c**) Loading and unloading profiles for a representative test.

While post-deformed measurements highlighted the pinning effect of O impurities, the real-time mechanical and microstructural response of individual nanograins (and GBs) subjected to the local stress field near an indent has yet to be elucidated, especially on a quantitative level. To this end, indentation tests were performed *in situ* inside a TEM (JEOL 2010F) using a Hysitron PicoIndenter (PI 95)^34^ on the NC Al films deposited on Si wedges. As shown in Fig. 2a,b, the film on the apex of wedge was transparent to the electron beam^31^,^32^, and was aligned to the axis of a diamond cube-corner tip (see more details of experimental set-up in Supplementary Fig. 8). Indentation was carried out in a displacement-controlled manner with simultaneous force measurement and dark-field TEM image acquisition, enabling a direct correlation between mechanical response and microstructural evolution beneath the indenter.

**Figure 3:**
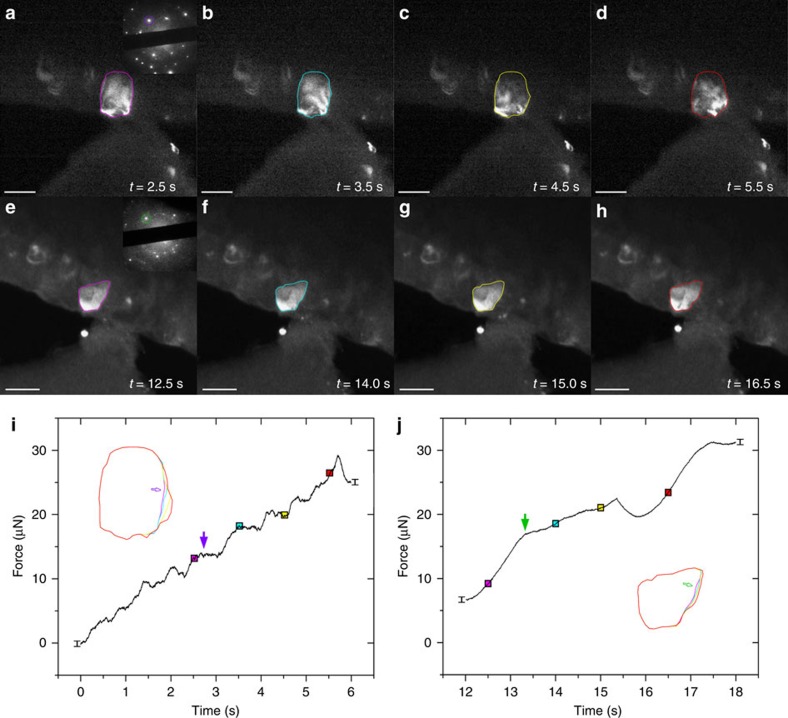
*In situ* TEM indentation tests of NC Al films showing mechanically driven GB migration. (**a**–**d**) Time-series of snapshots recorded during test of the film with *C*_O_ of 0.7 at.%. Inset: selected-area diffraction pattern of the film, with the outlined Al(111) spot used for dark-field TEM imaging. Scale bars, 100 nm. (**i**) Force–time curve. Vertical error bars show the peak-to-peak noise (∼1 μN) in force. Solid squares correspond to the snapshots shown in **a**–**d**. Solid arrow shows the onset of stress-driven GB migration. Inset: shape evolution of the grain outlined in **a**–**d**. GB migration only occurs in regions near the indenter. Open arrow shows the ‘hotspot' where GB migration was initiated. (**e**–**h**,**j**) Corresponding results of the film with *C*_O_ of 2.1 at.%. GB migration is still observed albeit to a lesser extent and initiated at higher forces. Scale bars, 100 nm.

Figure 3a–d represent a series of snapshots recorded during *in situ* indentation test of NC Al film with *C*_O_ of 0.7 at.%. A columnar grain is highlighted in dark-field images using individual diffraction spots in Al(111) and Al(200) rings (see inset of Fig. 3a), and Fig. 3i shows shape evolution of this grain and the corresponding force–time response, from which a few conclusions can be drawn. First, grain growth was only initiated when the applied force reached a critical value, and the onset of grain growth, occurring between frames 3a and b, corresponded to a measurable plateau in the real-time force signal (Fig. 3i). This correlation between the change in mechanical response and the onset of microstructural evolution is indicative of the stress-driven character of grain growth and its contribution to the incipient plastic deformation of NC metals. Second, grain growth was unambiguously observed with applied force beyond the critical value, although in some instances this occurred in conjunction with^35^,^36^ or subsequent to^37^,^38^ intragranular dislocation activities (as discussed later). Importantly, only the GB on the right side of the grain, which was closer to the indenter and thus undergoing higher stress, was found to be mobile and was subsequently driven towards the indenter, that is, the region with even higher stress. No detectable changes to the grain shape opposite to the indenter tip owing to GB migration or dislocation-mediated offsets were observed. We note that mobile GBs were always driven towards regions with higher shear stress, which is consistent with the shear-coupled mechanisms of GB migration^25^,^39^. However, one notable difference of our indentation tests with the models based on uniform shear stress is the presence of large stress gradients, highlighting the role of localized inhomogeneity of driving force in governing the progression from individual GB migration to discontinuous grain growth^40^,^41^. Finally, we observed that GB migration was not uniformly driven along the entire GB, but always initiated at some local hotspot, followed by bowing out of the neighbouring GB segment (see inset of Fig. 3i). Thus, the critical stress for GB migration can be defined according to the stress field at the hotspot, which reflects the local propensity for GB migration associated with the effectiveness of GB pinning, for example, by O impurities.

Addition of O impurities in the films mediated the onset of GB migration. Figure 3e–h,j represents another test of a NC Al film with *C*_O_ of 2.1 at.%. Similar characteristics of stress-driven grain growth and GB migration were observed, including (1) a critical force and concomitant yielding, (2) GB migration driven by (and towards) higher shear stress and consequent grain growth and (3) spatially inhomogeneous GB migration. However, the extent of GB migration was much less pronounced due to the increase of *C*_O_, which qualitatively supports the post-deformed measurements shown above.

### GB kinetics mediated by impurities

**Figure 4:**
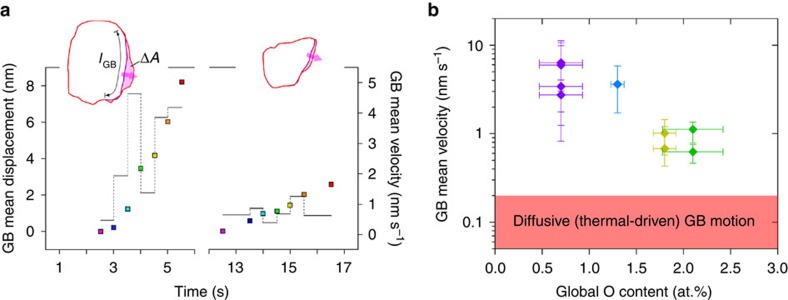
Retardation of GB kinetics by impurities. (**a**) Direct measurements of GB displacements (colour squares, left axis) and velocities (black lines, right axis) over time during *in situ* TEM indentation tests of the films with *C*_O_=0.7 at.% (left panel) and 2.1 at.% (right panel). The effective displacements at each snapshot relative to the pre-deformed state are approximated by the area swept by the moving GB (Δ*A*) normalized by the original GB length (*l*_GB_), and the mean velocities between consecutive snapshots reveal GB migration in a jerky manner. (**b**) Dependence of GB mean velocities (averaged over each indentation test) on *C*_O_ showing a significant retardation of GB migration owing to impurity pinning. Error bars of GB mean velocities for each test are determined as the s.d. of GB velocities shown in **a**. GB velocities representative of thermal-driven GB migration that is of a diffusive nature are shown in shaded region^24^, and are substantially lower than our measurements of stress-driven GB migration (over two orders of magnitude in the pure films).

These *in situ* indentation tests then enable GB-specific quantification of the characteristic parameters describing the mechanics and kinetics of stress-driven GB migration. We first quantify the velocity of GB migration by tracking the shape evolution of grains with applied force beyond the critical value. For simplicity, the effective GB displacement at each recorded snapshot (for example, Fig. 3a–h) was estimated as


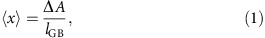


where *l*_GB_ is the original projected length of the mobile segment of GB and Δ*A* is the projected area swept by GB (Fig. 4a). The mean velocity of GB migration between each consecutive snapshots was thus calculated, and Fig. 4a shows GB migration to proceed in a jerky manner, which could be attributed to the spatially inhomogeneous de-pinning from impurities^29^. While quantitative modelling of GB kinetics and evaluation of GB mobility under various driving forces needs further theoretical efforts, a pronounced drag effect by (segregated) impurities is nonetheless revealed for the GB mean velocities averaged over each indentation test. As shown in Fig. 4b, GB mean velocity was decreased by ∼1 order of magnitude with *C*_O_ increasing from 0.7 to 2.1 at.%. The very fast GB migration in the purest films, with mean velocities up to 5–10 nm s^−1^, agreed well with previous *in situ* TEM tests of pure NC Al films^24^,^42^, in which grain growth and GB migration were activated by the high stress near crack tips. On the other hand, even the most retarded GB migration in the relatively impure films, with mean velocities of 0.7–1 nm s^−1^, were still significantly higher than the upper limit of GB velocity in thermal-driven GB migration, typically 0.2 nm s^−1^ for high-angle GBs and as small as 2 × 10^−5^ nm s^−1^ for low-angle GBs^24^,^42^,^43^. Therefore, we infer that the GB migration observed in our experiments was not (dominantly) carried out by atomic diffusion, but instead by the stress-driven collective motion of GB structural units, consistent again with the shear-coupled mechanisms^25^,^26^.

### Quantification of the critical shear stress for GB migration

**Figure 5:**
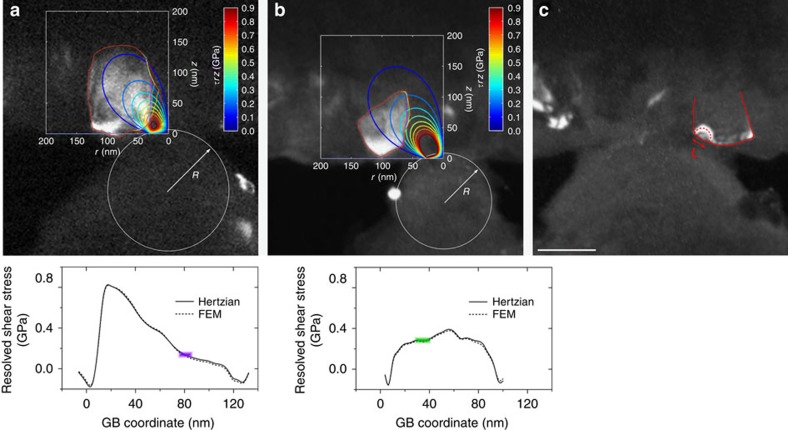
Quantification of impurity-mediated critical shear stress for GB migration and other deformation mechanisms. (**a**,**b**) Snapshots at the onset of GB migration in the films with *C*_O_ of (**a**) 0.7 and (**b**) 2.1 at.%, superimposed with subsequent shape evolution of the grains and contour maps of shear stress (*τ*_*rz*_) calculated using a Hertzian model (see text for details). Lower panels show corresponding profiles of resolved shear stress along the mobile GBs (both occurring on the right side of grains nearest to the indenter tip). Shaded bars denote the location of the onset of GB migration and estimated range of critical shear stress. (**c**) Snapshot of a different experiment showing the emission of dislocation (dash line) from a GB or triple junction (solid line). Scale bar, 100 nm. GBs were generally found to be immobile in cases where clear evidence of dislocation propagation was observed, indicating an alternative relaxation mechanism.

Now we turn our focus to the critical driving force (that is, shear stress) for GB migration. As a first-order approximation neglecting elastic anisotropy (expected to be small in Al (ref. 21)) and any plasticity before GB migration, the stress field in the vicinity of the indenter was estimated using the Hertzian model of elastic contact^44^, which described shallow indentation on a homogeneous half-space. The magnitude of shear stress at coordinate (*r*, *z*), as mapped in Fig. 5a,b, was calculated based on^45^:


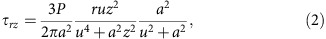


where 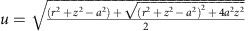, 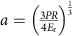 is contact radius, *P* is the critical force at the onset of GB migration, *R* is radius of curvature of the indenter as directly measured from TEM images and 
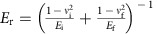
 is the reduced modulus derived from the modulus (*E*) and Poisson's ratio (*ν*) of indenter (i) and Al film (f). The influence of the wedge-shaped Si substrate on the stress distribution in the Al film was further examined using finite-element modelling. More details of modelling and error analysis of shear stresses are described in Supplementary Figs 9–12. Taken as a whole, the Hertzian model was proved to be a reasonable approximation for our experimental configuration to evaluate the stress field leading to the onset of GB migration. We note, though, that the modelling of the stress field during GB migration remains a challenge owing to limited knowledge of the stress relaxation mechanisms at dynamic GBs.

### Role of GB chemistry on mechanical stability

**Figure 6:**
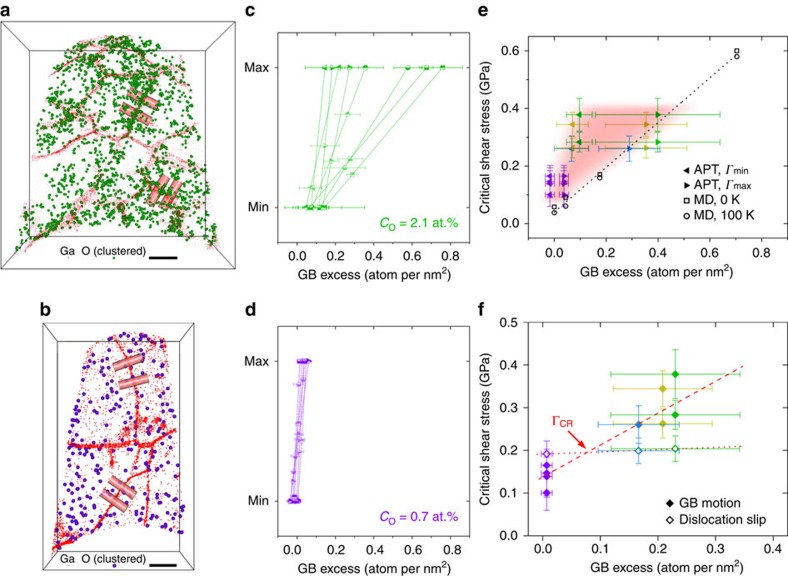
Quantification of GB excess of O atoms (Γ_O_) using APT and its role on mechanical stability of NC Al films. (**a**,**b**) APT reconstructions of films with *C*_O_ of (**a**) 2.1 and (**b**) 0.7 at.% showing the spatial distribution of O-rich clusters relative to GBs, highlighted by segregated Ga implanted during atom-probe sample preparation. Scale bars, 20 nm. (**c**) Variation of the areal density of O-rich clusters (Γ_O_) in the films with *C*_O_=2.1 at.% showing large inhomogeneity within a given GB (shown by a single line bounded by the minimum and maximum Γ_O_ values), as well as between different GBs (shown by different lines). Error bars of Γ_O_ are determined with the ‘cylinder method' (Supplementary Fig. 2). (**d**) A much narrower distribution of Γ_O_ was measured in the film with *C*_O_=0.7 at.%. Four representative analysed regions, that is, ‘cylinders' for such quantification are indicated in **a** and **b**. (**e**) Correlation between critical shear stress (*τ*_CR_) of GB migration and Γ_O_ averaged over all GBs measured by APT. Error bars of *τ*_CR_ are determined as described in Supplementary Figs 11 and 12. Error bars of Γ_O_ reflect the inhomogeneity between different GBs, and the ranges between local minima (left triangles) and local maxima (right triangles) of Γ_O_ reflect the inhomogeneity within individual GBs. Atomistic simulations^29^ of an Al bicrystal doped with O is shown (open symbols, dotted line) for comparison. (**f**) Full map of correlation between averaged GB excess and *τ*_CR_ for GB migration (solid symbols, linear fitting shown as dashed line) and dislocation propagation (open symbols, linear fitting shown as dotted line). The crossover of these two lines denotes a critical GB excess (Γ_CR_) for a transition of deformation mechanism that maintain NC microstructural stability under mechanical loads.

The critical shear stress (*τ*_CR_) for GB migration was consequently determined as the resolved shear stress along the mobile GBs, calculated from full stress fields (*τ*_*rz*_, *τ*_*rr*_ and *τ*_*zz*_) given by the Hertzian model^45^, at the hotspot of GB migration (Fig. 5a,b). Interestingly, GB migration was not necessarily initiated at the GB segment experiencing the highest resolved shear stress. This suggests either microstructural constraint (for example, immobile GB triple junctions^46^,^47^ and α-Al_2_O_3_ precipitates^33^) or a spatial variation of pinning strength within individual GBs associated with the inhomogeneous segregation of impurities. Herein, we focus attention on the O-rich clusters (with averaged sizes of 25–35 O atoms) segregated along GBs, which have been observed in APT characterization of the as-deposited films^33^ and are expected to dominate the GB pinning strength, though details of their inhomogeneous distribution have yet to be quantified. Figure 6a,b shows the spatial distribution of O-rich clusters in the films with *C*_O_ of 2.1 and 0.7 at.%, respectively. While the O-rich clusters were generally associated with GB regions, their area density, that is, the GB excess of O impurities (Γ_O_), varied not only globally (between different GBs) but also locally (within individual GBs), as quantified in Fig. 6c,d. More details of the ‘cylinder method' to calculate Γ_O_ are described in Supplementary Fig. 2.

Interestingly, two groups of GBs with distinct distributions of Γ_O_ were revealed in the impure films (Fig. 6c and Supplementary Fig. 3): one with a large difference between the minimum and maximum Γ_O_, and another with a relatively narrow distribution of Γ_O_. These distinct distributions of Γ_O_ presumably reflect the result of different GB structures that accommodate different amounts of impurities^48^, and can be rationalized by considering that the diameter of analysed regions (6 nm) was on the same order of the mean effective size of O-rich clusters (4–5 nm), but considerably smaller than the mean distance between clusters (10–15 nm), as revealed by previous APT analysis^33^. As a consequence, Γ_O_ would be insensitive to the position of analysed regions for the GBs only containing randomly distributed O solutes (such as low-angle GBs and ideal coincident site lattice GBs^48^), whereas GBs containing segregated O-rich clusters (such as random high-angle GBs and coincident site lattice GBs with larger deviations^48^) would show a wider range of Γ_O_ depending on the position of analysed regions relative to the discrete clusters. The inhomogeneity of Γ_O_ within such cluster-segregated GBs can be striking, in the extreme case ranging from ∼0.1 to ∼0.7 atom per nm^2^. By contrast, the distribution of Γ_O_ in the purest films (Fig. 6d), both between different GBs and within individual GBs, was found to be much narrower. Whereas *τ*_CR_ required for the onset of GB migration is governed by the interplay between the inhomogeneous distribution of Γ_O_ and the localized driving force, we note that confinement of microstructural features (for example, triple junctions^46^,^47^) may also contribute to the spatial inhomogeneity of GB pinning strength, while a higher extent of impurity segregation was expected near these features^23^. On the other hand, the effect of grain size alone is not considered to be prevalent, since the characteristic length scale of the spatial variation of Γ_O_ was remarkably smaller than grain sizes in the studied NC Al films. Taken as a whole, it is reasonable to examine the correlation between *τ*_CR_ and the full statistics of Γ_O_.

As shown in Fig. 6e, we find a positive correlation between our measurements of *τ*_CR_ (from *in situ* TEM indentation) and Γ_O_ (from APT analysis) in several NC Al films with *C*_O_ ranging from 0.7 to 2.1 at.%. This trend is robust to the specific quantity of Γ_O_ that was extracted from the statistical analysis of a variety of GBs, as both the minimum and maximum Γ_O_ within individual GBs generally increased with *C*_O_. Despite relatively dilute O concentrations in the films studied (and correspondingly small Γ_O_ values below 1 atom per nm^2^), we measure nearly a fourfold increase in *τ*_CR_ over the range of *C*_O_. Our results show qualitatively similar trends with atomistic simulations of a Σ75 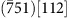 symmetric-tilt Al GB (ref. 29) showing a linear dependence of *τ*_CR_ for shear-coupled GB migration on the GB excess of O solute atoms (dotted line in Fig. 6e), despite the large differences in temperature, applied strain rate and GB characters. We also recognize the fact that the mechanical response of GBs vary with their crystallographic characters, as well as local chemical, structural and topological environment^48^. Nonetheless, the vast majority of GBs in our NC Al films, irrespective of *C*_O_, were found to be random high-angle GBs (see Supplementary Figs 4 and 5 and Supplementary Table 1 for more detailed characterization and statistics of GB crystallography), which preclude any simple correlation between GB excess and GB characters. This suggests that the detailed crystallography of GBs may serve only a secondary role in controlling GB migration in NC alloys, in contrast with pure materials. Thereby, the predominant role of segregated impurities in pinning GB migration is unequivocally revealed in our experiments, which represent a real NC system with complex GB networks.

## Discussion

Our experiments demonstrate that the critical stress for mechanically driven GB migration and its associated kinetics are primarily controlled by impurities, in our case O-rich clusters, that segregate to GBs. In light of this, we propose a transition in the governing deformation mechanisms in NC metals at a critical value of GB excess (Γ_CR_), which ultimately determines whether GBs (and thus NC microstructures) are stable under mechanical loads. Since *τ*_CR_ for stress-driven GB migration steadily increase with Γ_O_ (Fig. 6f, mean GB excess values were used for simplicity), it is likely to surpass the driving force for conventional dislocation activities beyond Γ_CR_. In these instances, the GBs are strongly pinned (in analogy to a Cottrell pinning atmosphere for dislocations) and plastic deformation would thereby need to be accommodated by propagation (that is, pinning and de-pinning) of discrete dislocations along GB ledges, a mechanism known to be operative and rate-limiting in ‘static' NC materials where intragranular dislocation interactions are abated^49^,^50^. Indeed, such dislocation activity in mechanically stable grains was observed on multiple occasions in our experiments (for example, Fig. 5c) and over the full range of *C*_O_ from 0.7 to 2.1 at.%. The shear stress to propagate a dislocation along a GB was then estimated using the simple relationship: *τ*_CR_=*Gb*/*L*,(3)where *G* is shear modulus, *b* is Burgers vector and *L* is the minimum diameter of the bowing dislocation segment observed before de-pinned from GB pinning points^24^. We note that intragranular dislocation activities did occur in mechanically unstable grains, nonetheless, the onset of stress-driven microstructural evolution was unambiguously defined by the *τ*_CR_ for GB migration. Combining both the mechanically dynamic and static GBs thereby provides a full map of *τ*_CR_ for incipient plastic deformation versus Γ_O_ (Fig. 6f), revealing an apparent crossover at Γ_CR_∼0.1 atom per nm^2^.

This delineation between mechanically dynamic and static NC microstructures, as controlled by the impurity content (and GB excess), qualitatively agrees with previous micro-tension tests of NC Al films that showed suppression of mechanical grain growth owing to GB impurities, yet where only global information was available^23^. Observing that the macroscopic response of NC materials is determined by averaging over the ensemble of grains with widespread GB characters and orientations of slip systems, the crossover of *τ*_CR_ with increasing Γ_O_ indicates a transition of the most probable deformation mechanism from GB migration to dislocation propagation. This new insight helps address the reported transition of micro-tensile behaviour of NC Al films from ‘ductile and moderately strong' to ‘very strong but brittle'^23^. More importantly, the highly localized stress field generated by *in situ* indentation tests enabled us to discover all branches of deformation mechanisms shown in Fig. 6f provided that the individual grain and GB were of proper orientation, even though dislocation slip in pure films and GB migration in impure films were statistically unfavourable.

The striking implication from our *in situ* measurements and analysis is twofold. First, the critical stress for the promotion of GB migration, the precursor for widespread mechanical grain growth in NC materials, can be measured at the individual GB level, and the value of this stress can be modulated by processing with alloying species that segregate to GBs. Comparing the degree of alloying required for thermal stability with that of mechanical stability, and examining the atomic mechanisms responsible for thermodynamic stability and retarded kinetics would be an interesting avenue for future pursuits. Second, our results suggest a crossover between dynamic and static deformation mechanisms, and consequently a tunable mechanical stability of NC materials, with only a dilute concentration of alloying species. This enables the wide spectrum of materials design with minimal constraints of materials costs and abundance. Most tantalizing, though, is the notion of a new structural materials design strategy that offers high strength paired with thermal and mechanical stability, juxtaposed with adaptive deformation mechanisms that can respond to locally high stresses in extreme conditions in a self-healing fashion.

## Methods

### Synthesis of NC Al films

NC Al films studied in our *ex situ* and *in situ* indentation tests were synthesized by magnetron co-sputtering of a 99.999% pure Al target and a 99.995% pure α-Al_2_O_3_ target with a confocal geometry^33^. The direct-current (d.c.) power of Al target was varied from 200 to 400 W, and the radio-frequency (RF) power of α-Al_2_O_3_ target was varied from 0 to 50 W. The as-deposited films manifested a composite-like microstructure^33^ containing O impurities with distinct morphologies, that is, α-Al_2_O_3_ precipitates, O-rich clusters and O solute atoms.

### *Ex situ* and *in situ* nanoindentation experiments

The *ex situ* nanoindentation experiments were performed using a Hysitron TriboIndenter (TI 950) employing a diamond Berkovich tip, with an indentation strain rate of 0.05 s^−1^ and maximum indentation depth of ∼0.5*t*. Additional indentation tests were performed *in situ* inside a TEM (JEOL 2010F) using a Hysitron PicoIndenter (PI 95)^34^ on the NC Al films deposited on Si wedges. Indentation was carried out with a constant rate of 5 nm s^−1^ applied in step-wise loading segments (Fig. 2c), while force was simultaneously measured with a peak-to-peak noise level of 1 μN.

### Atom-probe tomography and data analysis

The same atom-probe data sets as reported in ref. 33 were utilized for the calculation of GB excess using ‘cylinder method'. Briefly, atom-probe specimens were prepared inside a Zeiss Auriga FIB/SEM by a lift-out approach^51^. Atom-probe data were acquired using a Cameca LEAP 4000X Si operated in voltage-pulsing mode at 40 K, with pulsing voltage being 20% of d.c. bias voltage, and evaporation rate kept at 1% of the pulsing rate of 200 kHz (ref. 33). Data reconstruction and visualization were performed using IVAS software (Cameca). O^+^, AlO^+^ and minor amount of O_2_^+^, O^2+^, AlO^2+^, AlO_2_^+^ and AlO_2_^2+^ were identified in the mass spectrum as O-containing species. Analysis of O-rich clusters was facilitated by means of Voronoi volume distribution through custom MATLAB (Mathworks Inc.) programs.^52^

## Additional information

**How to cite this article:** He, M.-R. *et al*. Linking stress-driven microstructural evolution in nanocrystalline aluminium with grain boundary doping of oxygen. *Nat. Commun.* 7:11225 doi: 10.1038/ncomms11225 (2016).

## Supplementary Material

Supplementary Information Supplementary Figures 1-12, Supplementary Table 1, Supplementary Notes 1-5 and Supplementary References.

Supplementary Movie 1 In situ dark-field TEM indentation experiments of NC Al films showing mechanically driven GB migration during test of the film with CO of 0.7 at. %. Right panel: Corresponding force-time curve during experiment, where the peak-to-peak noise in force is ~ 1 μN.

Supplementary Movie 2 In situ dark-field TEM indentation experiments of NC Al films showing mechanically driven GB migration during test of the film with CO of 2.1 at. %. Right panel: Corresponding force-time curve during experiment, where the peak-to-peak noise in force is ~ 1 μN.

Supplementary Movie 3 In situ dark-field TEM indentation experiment showing the emission of dislocation from a GB or triple junction. GBs were generally found to be immobile in cases where clear evidence of dislocation propagation was observed, indicating an alternative relaxation mechanism.
