# Supplementary material for: Linking stress-driven microstructural evolution in nanocrystalline aluminium with grain boundary doping of oxygen
Source: Nat Commun. 2016 Apr 13;7:11225. doi: 10.1038/ncomms11225 (PMC4833861; doi:10.1038/ncomms11225)
Supplement: Supplementary Information — Supplementary Figures 1-12, Supplementary Table 1, Supplementary Notes 1-5 and Supplementary References. [file ncomms11225-s1.pdf]

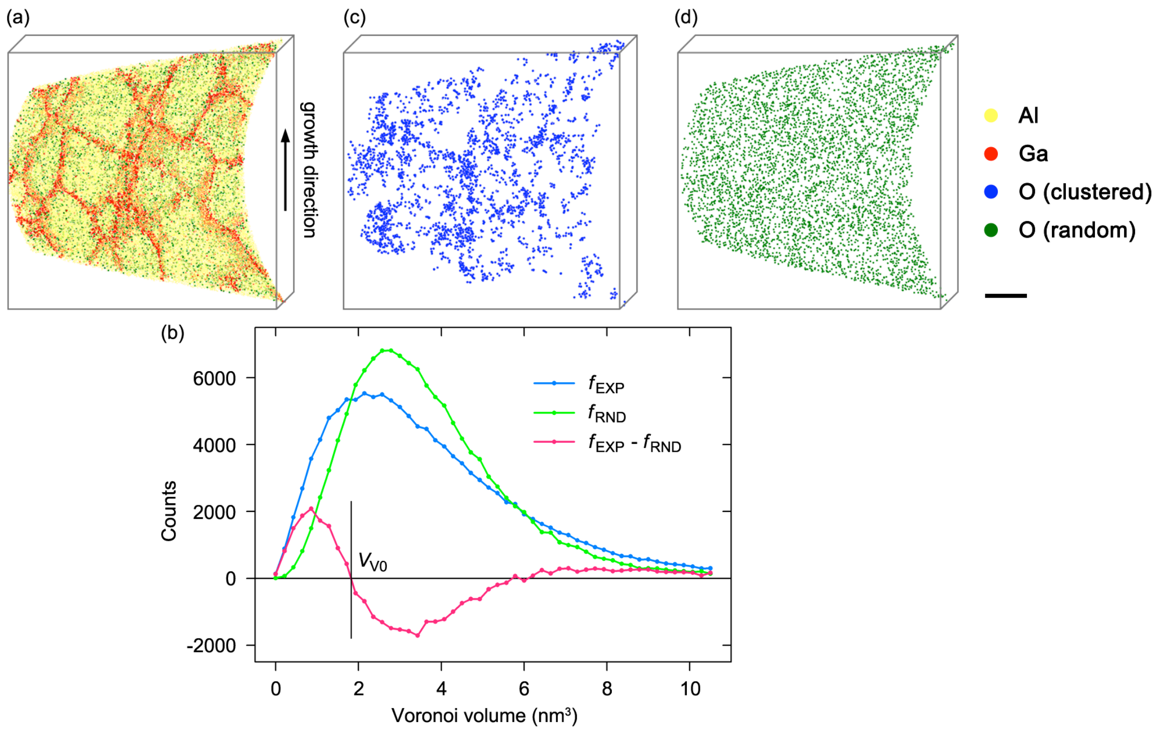

Supplementary Figure 1 | Atom-probe tomography (APT) characterization of nanocrystalline (NC) Al films. **(a)** Reconstructed image of a slice with volume of 130×120×8 nm^3 from the films with global O content (C_O) of 2.1 at. %. Al and Ga atoms are represented by yellow and red dots, respectively. **(b)** Experimental distribution of Voronoi volumes for all O atoms in the specimen (blue curve) and calculated distribution for the same number of randomly distributed O atoms (green curve). Their difference (red curve) defines the threshold (V_{V0}) between O-rich clusters and random solutes. **(c, d)** Spatial distribution of **(c)** O-rich clusters (blue dots) and **(d)** O solutes (green dots) in **(a)**. Scale bars: 20 nm.

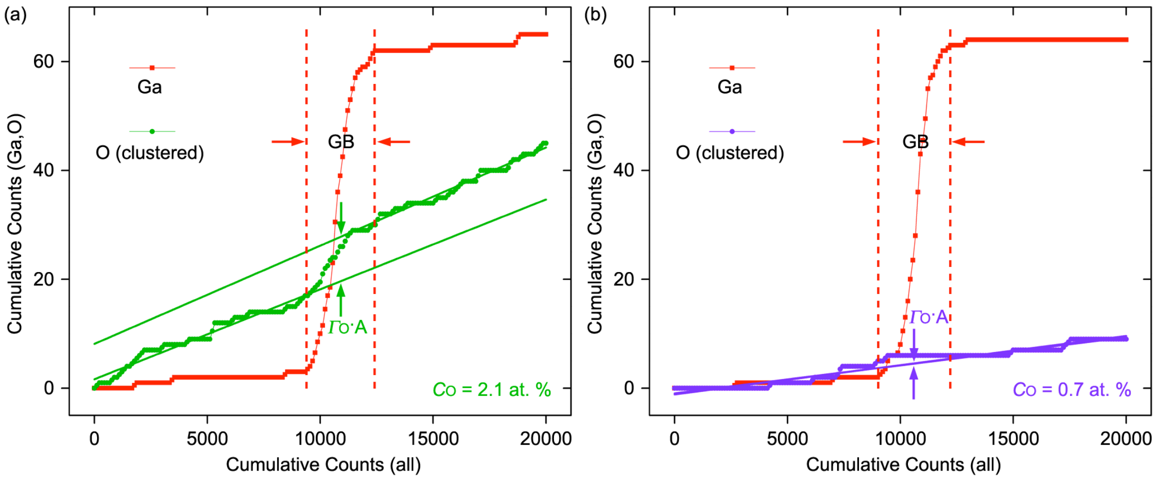

Supplementary Figure 2 | Calculation of grain boundary (GB) excess of O (Γ_O) using "cylinder method". The plot of cumulative number of clustered O atoms (green in **(a)**, purple in **(b)**, the detector efficiency has been accounted for) versus cumulative number of all atoms was linear-fitted within grain interiors on both sides of GB region defined by the segregated Ga atoms. The excess number of clustered O atoms ($\Xi_O = \Gamma_O \cdot A$) was then determined by the difference between intercepts of the two linear fits at a dividing plane defined by half of step height of the cumulative number of Ga atoms (red).

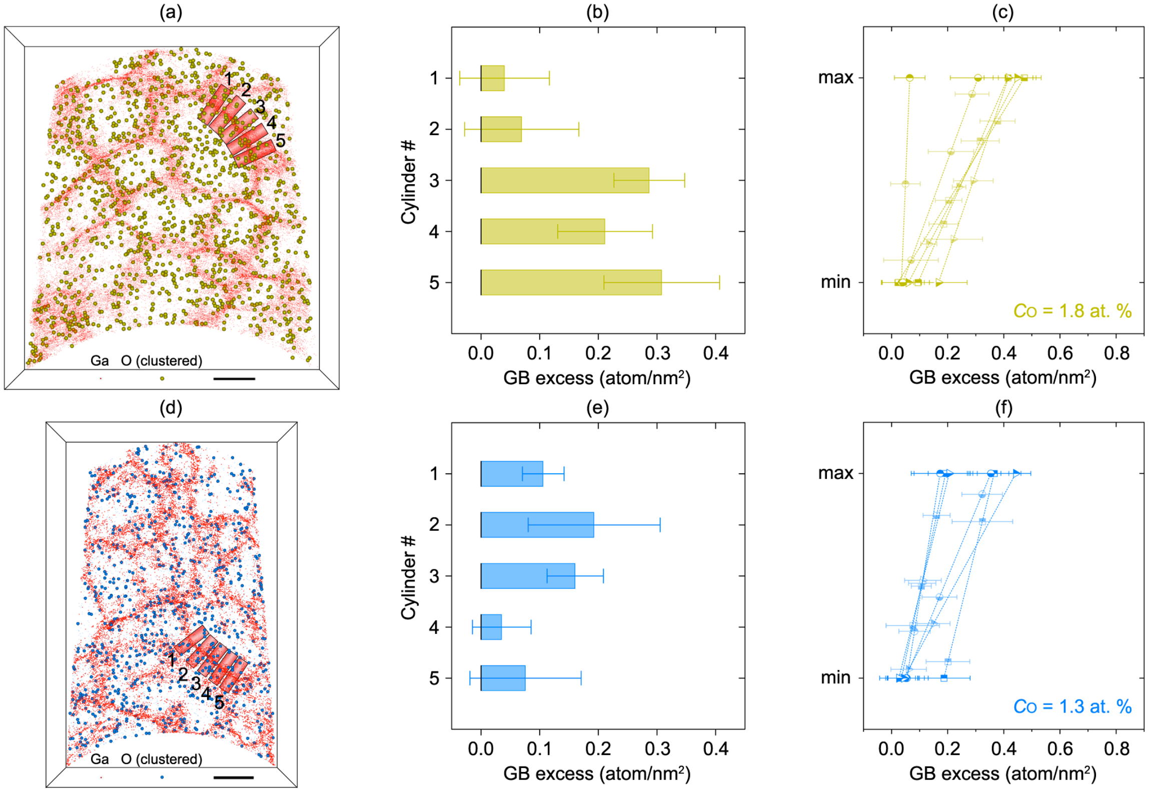

Supplementary Figure 3 | Inhomogeneous spatial distribution of O-rich clusters. (a) APT reconstruction of a film with C_O of 1.8 at. % showing the segregation of O-rich clusters along GBs. **(b)** Γ_O measured at different positions within an individual GB, shown by the multiple cylinders in **(a)**. **(c)** Variation of Γ_O in different GBs of the film. Each GB is shown by a single line bounded by the minimum and maximum Γ_O values measured in this GB, and other Γ_O values in this GB are linearly interpolated into the line. **(d-f)** Spatial variation of Γ_O measured in a film with C_O of 1.3 at. %. All error bars of Γ_O are determined by the "cylinder method". Scale bars: 20 nm.

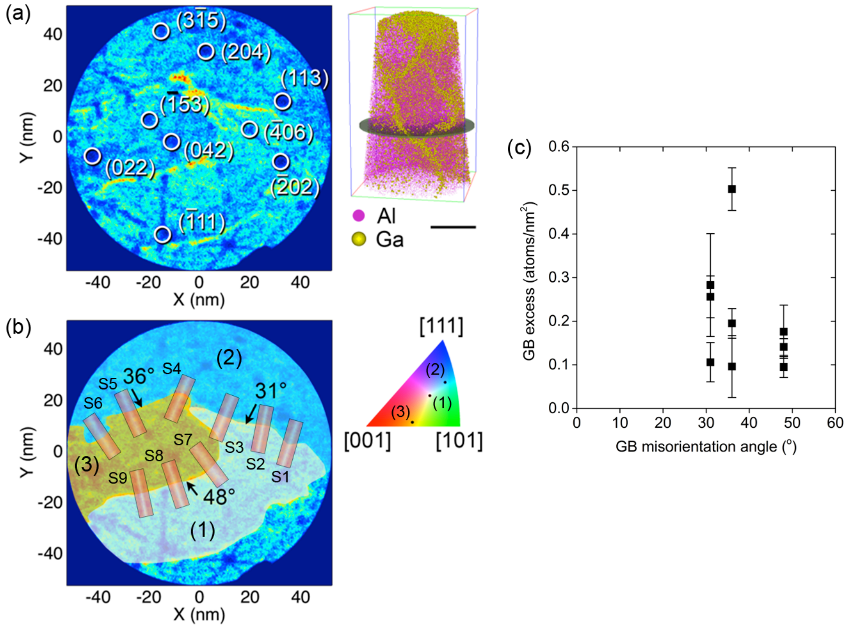

Supplementary Figure 4 | Crystallographic analysis of a representative atom probe dataset. **(a)** Density map of ~ 1 million atoms slice within three-dimensional reconstruction (Inset, locations of GBs are also outlined by the segregated Ga ions. Scale bar: 50 nm). Three poles in each grain are indexed. **(b)** Orientation map of indexed grains relative to the detector and misorientation angles between each grain. Cylinders S1~S9 show the positions where local GB planes and GB excess are measured (see Supplementary Table 1). **(c)** Correlation between excess value and misorientation angle of the GBs in **(b)**.

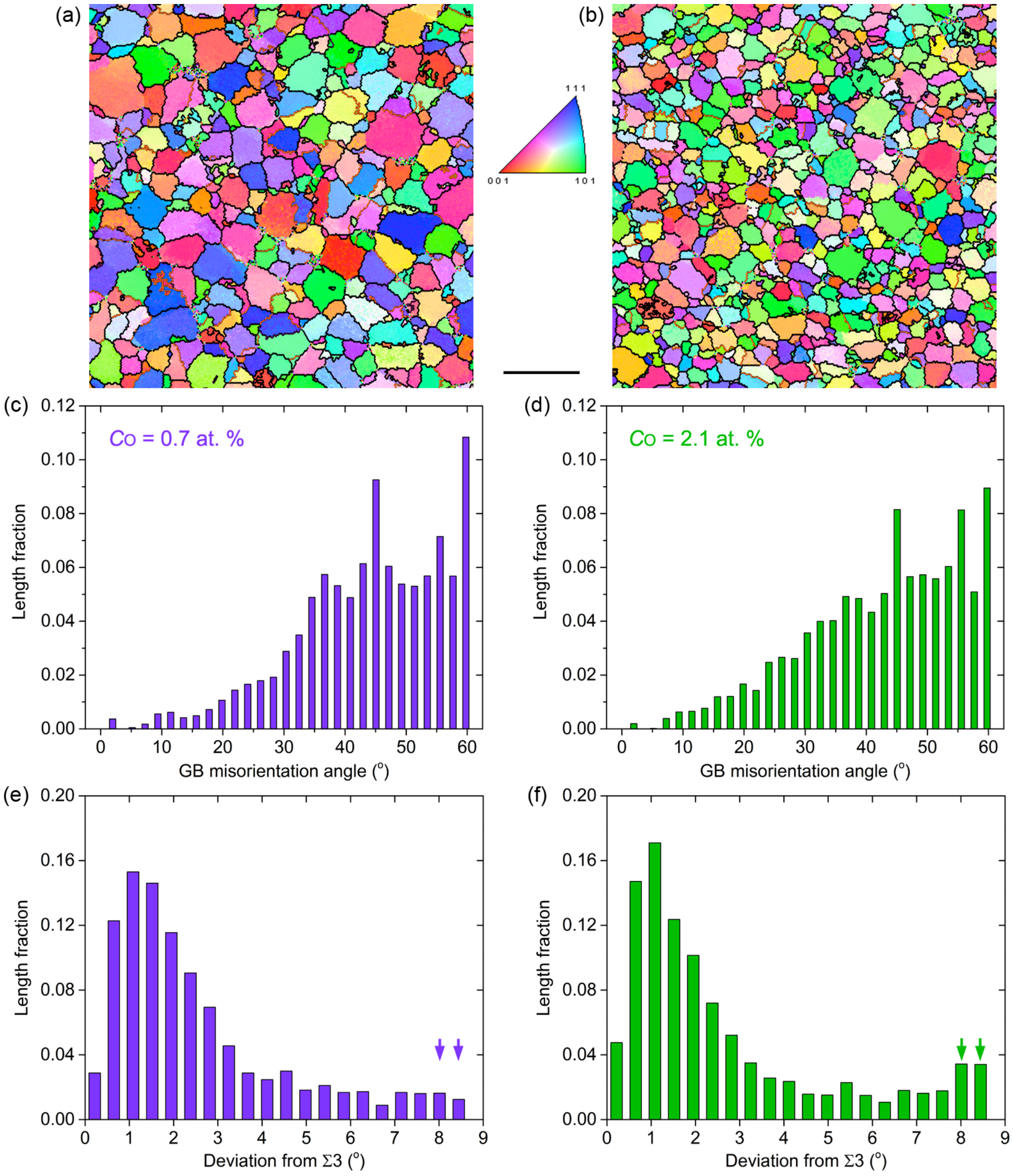

Supplementary Figure 5 | Statistical analysis of GB crystallography using Transmission Kikuchi Diffraction (TKD). (a) Out-of-plane orientation mapping of grains in the film with C_O = 0.7 at. %. **(c)** Distribution of GB misorientation angles. **(e)** Distribution of deviation angles from the ideal symmetric Σ3 misorientation. **(b, d, f)** Results of the film with C_O = 2.1 at. %. Arrows in **(e)** and **(f)** show GBs with large deviation. Scale bar: 200 nm.

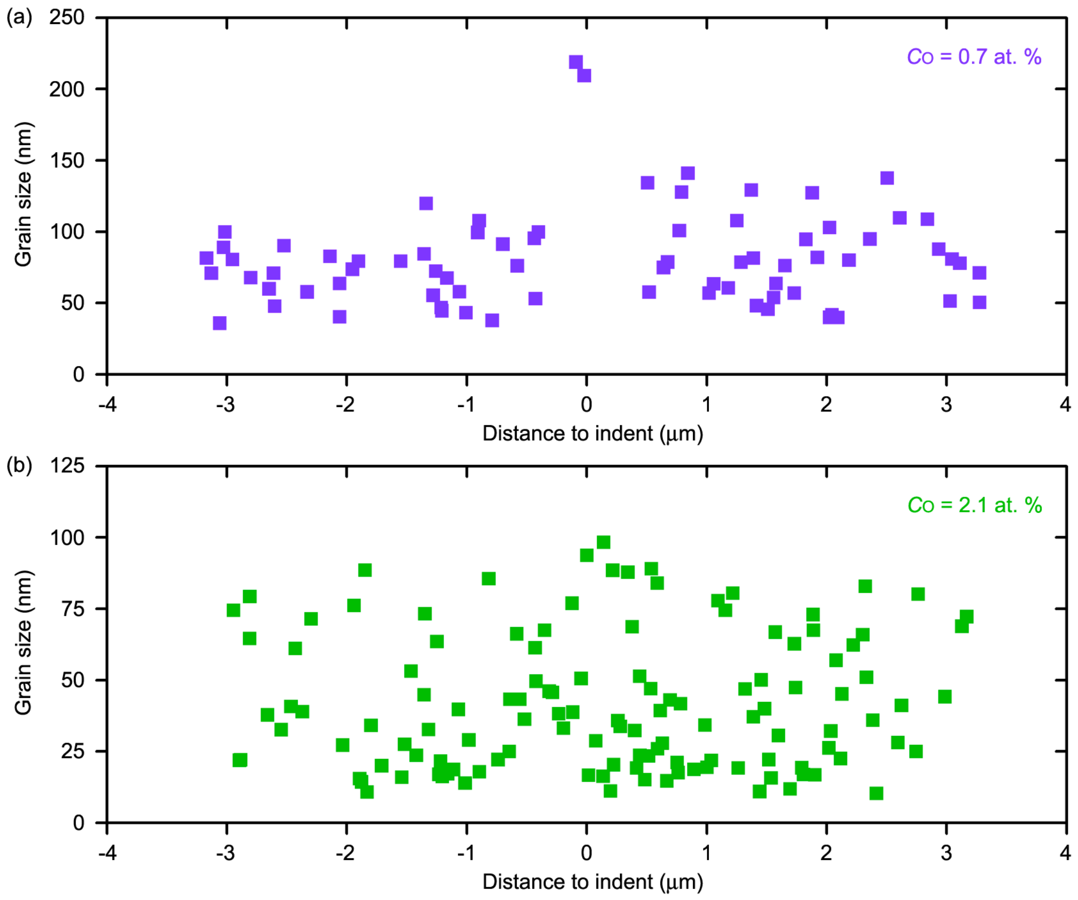

Supplementary Figure 6 | Grain size statistics in the post-deformed regions of NC Al films with C_O of **(a)** 0.7 at. % and **(b)** 2.1 at. % (see Fig. 1 in main text).

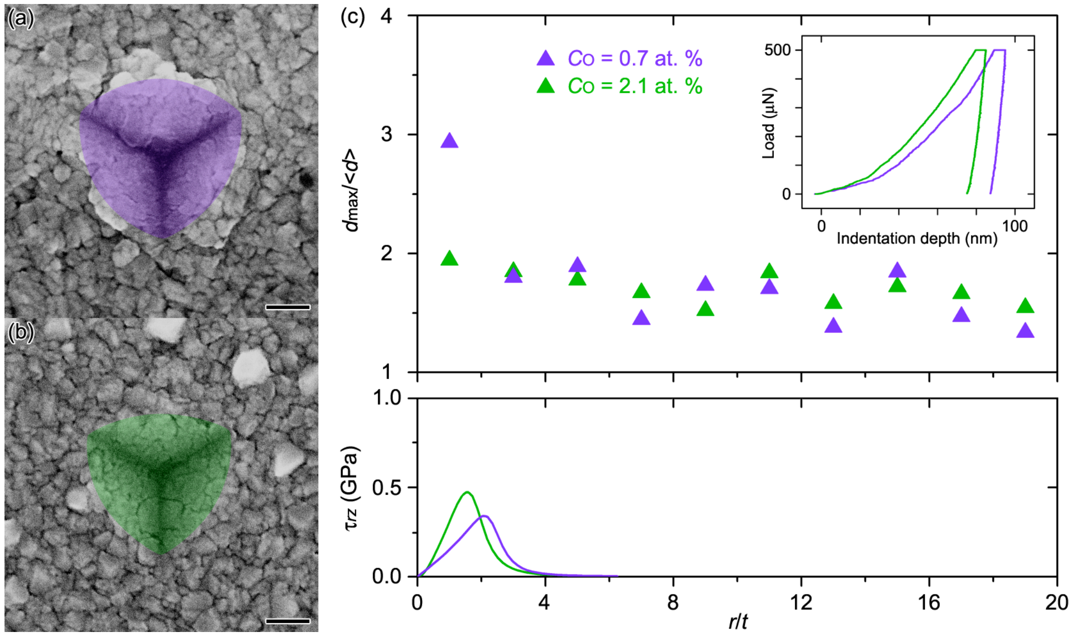

Supplementary Figure 7 | Estimation of stress field. (a, b) SEM images of indents in NC Al films with C_O of **(a)** 0.7 at. % and **(b)** 2.1 at. %. Contact areas are shown in color. Scale bars: 200 nm. **(c)** Distribution of shear stress near indents calculated using Hertzian model. The upper part is Fig. 1(e) in main text, showing impurity effect on stress-driven (discontinuous) grain growth.

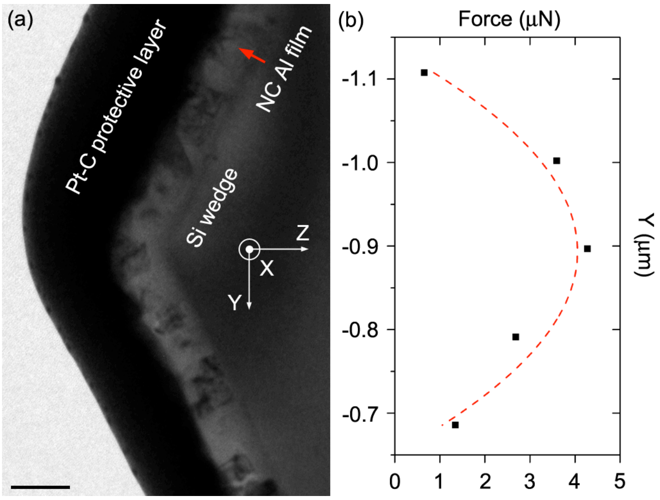

Supplementary Figure 8 | Alignment of indenter to the surface of film. (a) Cross-sectional TEM image of NC Al film on Si wedge. The outmost dark layer, which is Pt-C coated during preparation of cross-sectional specimen, does not exist during *in situ* TEM tests. Scale bar: 200 nm. **(b)** A representative tip alignment test. The peak of dash curve corresponds to the proper Y position for later indentation tests (the form of peak function was not specific).

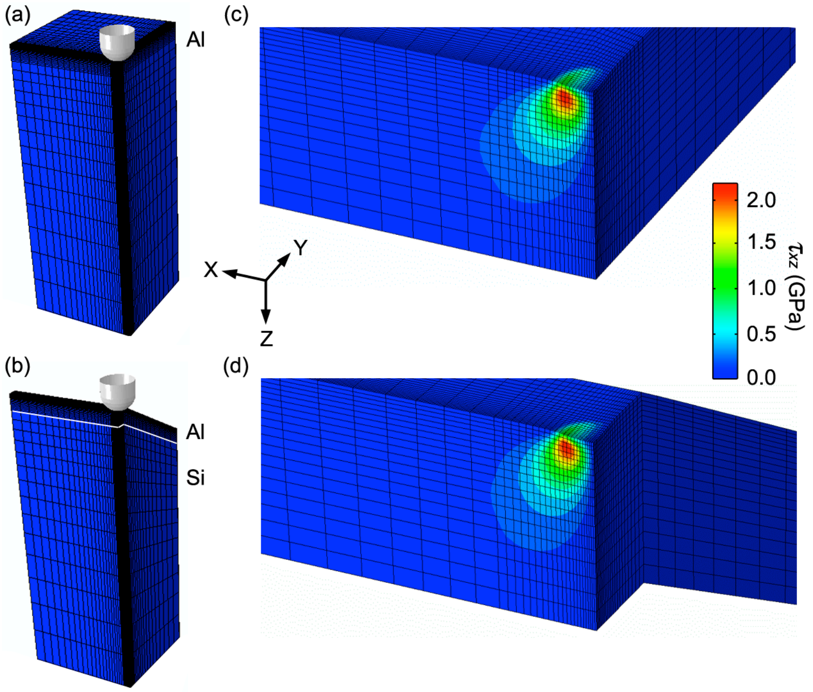

Supplementary Figure 9 | Finite element modeling (FEM) of indentation stress field. (a, b) The geometry and mesh configuration of **(a)** half-space as in Hertzian model and **(b)** film-on-wedge geometry of experimental tests. **(c, d)** Distribution of shear stress in Al film at maximum load of 13.7 μN.

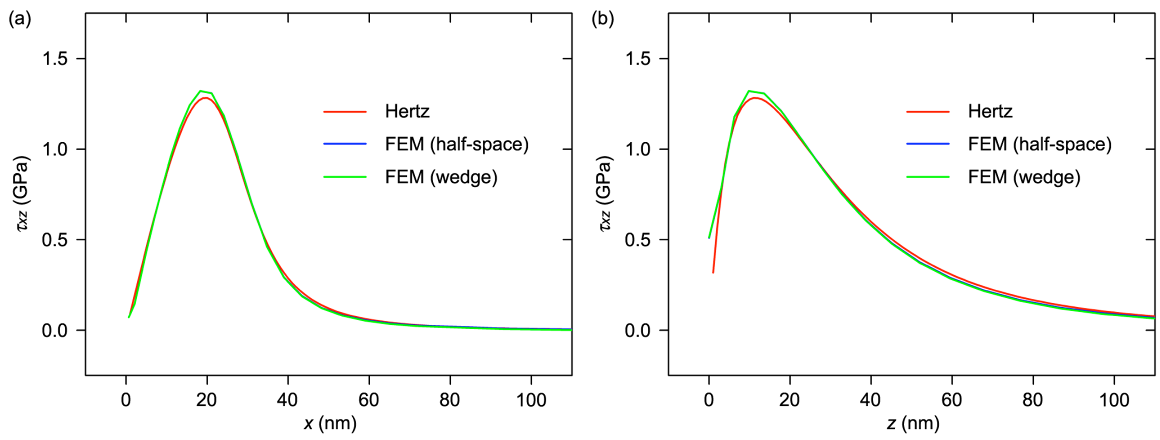

Supplementary Figure 10 | Across-maximum (a) *x*- and (b) *z*-profiles of shear stress. Red: analytical solution of Hertzian model; Blue: FEM of half-space; Green: FEM of film-on-wedge geometry. The accordance between Hertzian model and FEM results may be further improved by using higher mesh density.

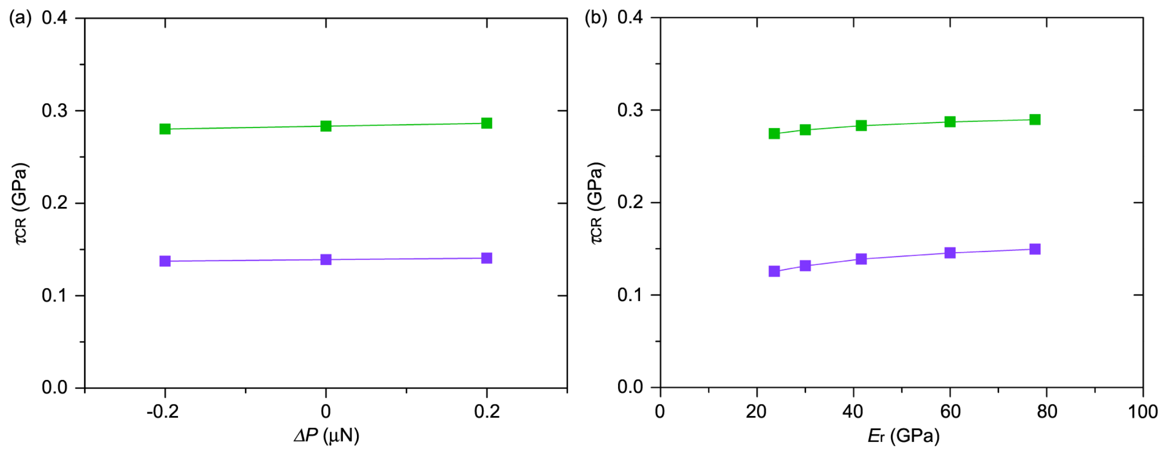

Supplementary Figure 11 | Variation of critical shear stress with **(a)** measurement error in critical force and **(b)** reduced modulus.

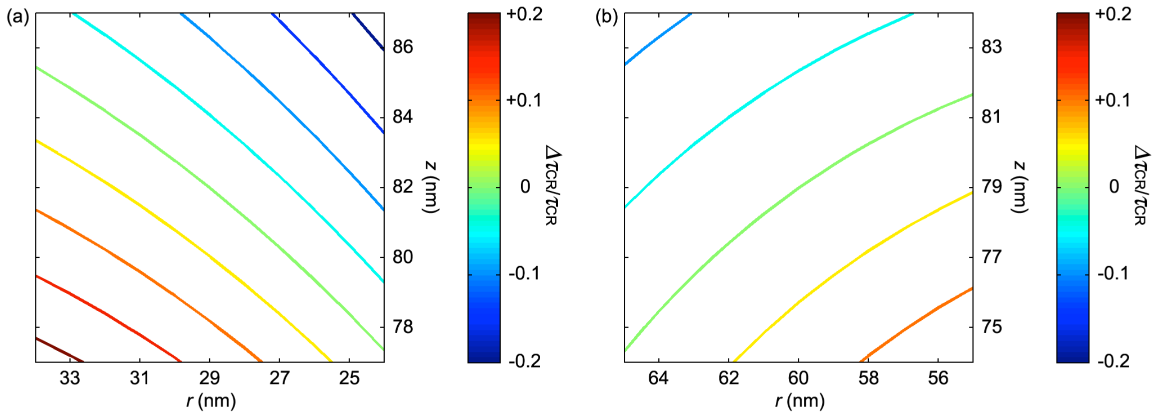

Supplementary Figure 12 | Variation of critical shear stress with coordinate. (a) and **(b)** correspond to Fig. 5(a) and 5(b) in main text, respectively.

Supplementary Table 1 | Complete crystallographic and chemical analysis of each GB in Supplementary Fig. 4(b).

Grain A	Grain B	GB misorientation angle / axis <*u v w*>	Site	GB plane A {*h k l*}	GB plane B {*h k l*}	$\boldsymbol{\Gamma}_{O}$ atom nm^{-2}
1 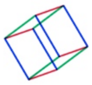	2 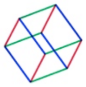	31.1°	S1	0.869 -0.028 0.495	-0.410 0.377 0.830	0.106
			S2	0.833 -0.020 0.553	-0.459 0.330 0.825	0.283
		-0.441 -0.896 -0.048	S3	0.869 -0.029 0.494	-0.409 0.377 0.831	0.266
2 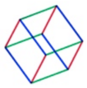	3 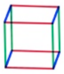	36.3°	S4	0.190 0.745 0.640	-0.327 0.732 0.597	0.503
			S5	-0.334 0.858 0.390	-0.421 0.221 0.880	0.106
		-0.377 0.429 0.821	S6	-0.778 0.608 0.159	-0.225 -0.301 0.927	0.195
1 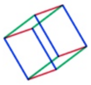	3 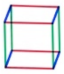	48.4°	S7	0.330 0.943 0.051	-0.583 -0.487 0.651	0.095
			S8	0.448 0.893 -0.039	-0.649 -0.347 0.677	0.141
		0.311 0.526 0.791	S9	0.468 0.883 0.037	-0.588 -0.356 0.726	0.176

Supplementary Note 1 | Microstructural characterization of NC Al films

As described in our previous work [1], the as-deposited NC Al films manifested a composite-like microstructure containing O impurities with distinct morphologies, i.e., α-Al_2O_3 precipitates, O-rich clusters, and O solute atoms, which showed sequentially decreasing pinning strengths against dislocation activities though increasing populations. Similarly, the precipitates would serve as strong but sparse obstacles against stress-driven microstructural evolution. Thus, the "hotspot" of GB motion, as observed during *in situ* indentation tests inside the TEM, should be present away from those precipitates.

Supplementary Fig. 1 shows representative APT characterization of the as-deposited NC Al films [1]. Al GBs were outlined by the segregated Ga atoms (Supplementary Fig. 1(a)), a well-understood artifact of specimen preparation process using a focused-ion beam [2], and the local clustering of O atoms was highlighted by means of Voronoi volume distribution analyses [3] (Supplementary Fig. 1(b)). We revealed that O-rich clusters (with averaged sizes of 25~35 O atoms) were mostly present in the vicinity of Al GBs (Supplementary Fig. 1(c)), while the remaining O solute atoms were uniformly dispersed throughout the film (Supplementary Fig. 1(d)). Furthermore, the number of clustered O atoms was approximately proportional to C_O over the range from 0.7 at. % to 2.1 at. % [1]. These O-rich clusters segregated along GBs are thus expected to play an important role in governing the inhomogeneous GB pinning field and the critical stress required for the onset of GB motion.

Given the distinct spatial distributions of O-rich clusters and solute atoms, Γ_O was determined by the area density of O-rich clusters, as calculated using the conventional "cylinder method" [4]. Briefly, 3~5 analyzed regions, i.e. "cylinders", were placed across each GB, and 6~8 GBs were analyzed for each atom probe dataset. The cylinders were 6 nm in diameter and 20 nm in length, with axis parallel with local normal of GBs. Fig. 6(a) and 6(b) of main text show some of these cylinders, and Supplementary Fig. 2 plots the cumulative number of clustered O atoms and GB-segregated Ga atoms versus the

cumulative number of all atoms in two representative cylinders from the films with C_O of 2.1 at. % and 0.7 at. %, respectively. We note that only 55 % of all atoms in any atom probe specimen were detected by APT methods, and this detector efficiency has been accounted for in our calculations. The local Γ_O value was thus defined as: $\Gamma_O = \Xi_O/A$ [4], where Ξ_O is the corrected excess number of clustered O atoms associated with the GB, i.e. the step height across GB, and $A = 9\pi$ nm^2 is the cross-sectional area of cylinders. Γ_O in the two cylinders shown in Supplementary Fig. 2(a) and 2(b) were calculated to be 0.289±0.031 atom/nm^2 and 0.006±0.015 atom/nm^2, respectively, which represent the distinct GB pinning strength as controlled by O impurities.

While O-rich clusters were generally located near GBs, the local Γ_O values measured in different GBs as well as different positions within individual GBs show remarkable variation, as demonstrated in Supplementary Fig. 3. The two groups of GBs showing distinct distributions of Γ_O, as in Fig. 6(c) of main text, were also noticed in the films with C_O of 1.8 at. % and 1.3 at. %. Overall, the impurity pinning against stress-driven microstructural evolution was confirmed to be inherently inhomogeneous, and is thought to depend on both crystallographic characters and local environments of GBs [5].

Furthermore, APT methods offer us the unique opportunity to study the relationship between GB characters (misorientation angle / axis, and GB plane) and GB segregation for each individual pairs of grains [6,7]. To demonstrate this process, an atom probe dataset from the film with C_O of 1.8 at. % was arbitrarily selected and was found to present sufficient information to calculate crystallography of several GBs. Supplementary Fig. 4(a) shows the density map of a slice (see inset) within three-dimensional reconstruction of the dataset. Higher density regions correspond to the location of GBs, whereas lower density regions reveal zone lines and poles containing crystallographic information of each grain, resembling a stereographic projection of the crystal orientations. These regions are known to have high (atomic) in-depth resolution that enables the detection and index of individual lattice planes by using Z-spatial distribution

maps. Three poles in each grain were indexed to calculate the grain orientation relative to the detector. In consequence, orientations of three grains were determined in this dataset, as shown in Supplementary Fig. 4(b) and inset, and each of the three GBs probed was found to be (random) high-angle boundary. In the meantime, Γ_O values were calculated across three different locations within each of the GBs by using the “cylinder method”. The average Γ_O for the three GBs shown in Supplementary Fig. 4(b), with misorientation angles of 31°, 36° and 48°, are 0.22, 0.27 and 0.13 atoms nm^{-2}, respectively, while the extent of segregation also varies considerably across single GBs, as shown in Supplementary Fig. 4(c). This is again consistent with previous APT observations [5] indicating that no simple correlation exists between GB excess and misorientation angle in the high-angle regime, and that local structural environment (such as GB planes) also play a decisive role in GB segregation of alloying species. We further show in Supplementary Table 1 that the local GB planes at which Γ_O values were calculated can also be determined using APT methods, though an explicit correlation between GB planes and GB excess may not be hitherto expected.

In addition, we carried out a statistical analysis of GB characters in the NC Al films with C_O = 0.7 at. % and 2.1 at. %, in order to glean more insight on the links between GB excess, GB crystallography, and the critical stress for GB motion. Crystallography of hundreds of NC grains and GBs were mapped by using TKD method [8,9] with spatial resolution far superior to the conventional electron backscattered diffraction (EBSD) methods. Briefly, the films were deposited on TEM grid with carbon films (alongside the Si(100) substrates and micro-machined Si wedges), and were mounted in SEM with tilted angle of 0°~15° between the film surface normal and incident beam. Grain orientations were then measured at an acceleration voltage of 30 kV using an EBSD system (Hikari camera, TSL software). Supplementary Fig. 5(a) and 5(b) show out-of-plane orientations of the grains comprising the films with C_O = 0.7 at. % and 2.1 at. %, respectively. No strong texture is observed in either sample, indicating that no systematic variation exists

in the crystallography of NC grains (and thus GBs) that are randomly sampled during our *in situ* indentation tests. Supplementary Fig. 5(c) and 5(d) further show the corresponding distributions of GB misorientation angles of the two films. We notice that the vast majority of GBs in our study, irrespective of film composition, are high-angle GBs (with misorientation > 15°), where a simple correlation between GB excess and misorientation angle (as observed in low-angle GBs) no longer exists [5]. Therefore, the chemically modulated critical stress for GB motion, as well as their associated velocities, can be attributed primarily to the difference in impurity content of the randomly sampled GBs.

Then, we focus attention on the relatively high fraction of coincident site lattice (CSL) boundaries with special orientations. For instance, the highest peak at 60º misorientation in Supplementary Fig. 5(c) and 5(d) represents Σ3{111} twin boundaries, which have much lower energy relative to other random high-angle and CSL GBs and thus allow for minimal solute segregation [5]. However, the most recent experiments by Herbig et al. revealed that solute excess in these GBs increases with the angular deviation from the ideal high-symmetry CSL misorientation, which can occur by the presence of GB facets, partial dislocations, and elastic strain fields [10,11]. In this regard, we focus on the GBs indexed as Σ3 type according to the Brandon criterion [12] in the two films with C_O = 0.7 at. % and 2.1 at. %, and calculated their distributions of deviation from the ideal symmetric Σ3 misorientation, as shown in Supplementary Fig. 5(e) and 5(f), respectively. With an increasing C_O, a notably increasing fraction of GBs are found to have larger deviation, suggesting that the CSL GBs in "impure" samples are even farther from equilibrium. Therefore, we conclude that the majority of GBs present in our NC films - even those CSL GBs - are expected to show increasing Γ_O with increasing C_O, independent of the specific crystallography of the GB.

Similar analysis of GB crystallography was also performed for the films deposited on Si wedges, as used for *in situ* TEM testing. While statistics in these samples are much more limited, the results are well supported by the full statistics of the plan-view samples,

namely, lack of strong texture and dominance of high-angle GBs in both films, and more GBs with large deviation from ideal Σ3 misorientation in the more impure films.

Taken as a whole, our microstructural characterization of the NC Al films suggest the following: (i) there is no statistically significant difference in the distributions of GB crystallography between the films with different C_O, and (ii) any dependence of the critical shear stress for GB motion and GB crystallography for the GBs studied here appears to be a direct manifestation of the dependence of the critical stress on Γ_O.

Supplementary Note 2 | Mechanical grain growth induced by instrumented nanoindentation

Supplementary Fig. 6 shows the grain size statistics measured in the cross-sectional specimens (see Fig. 1(a) and 1(b) of main text) of NC Al films after nanoindentation. All grains were manually traced out in dark-field TEM images. For each grain, the grain size was defined as the area-equivalent circular diameter, and the coordinate was defined as the lateral distance of its center-of-mass to the indent. Fig. 1(g) of main text was then obtained by lateral binning of these raw data and picking up the maximum grain size in each bin.

Above measurements indicate that (discontinuous) grain growth was driven by the higher stress field near the indent. To show this, the distribution of shear stress near the indent was calculated in both films, using Hertzian model of elastic contact [13]:

$$\tau_{rz}(r) = \frac{3P}{2\pi a^2}\frac{ruz^2}{u^4 + a^2z^2}\frac{a^2}{u^2 + a^2}, \qquad (1)$$

where $u = \sqrt{\frac{(r^2 + z^2 - a^2) + \sqrt{(r^2 + z^2 - a^2)^2 + 4a^2z^2}}{2}}$, r is the distance to indent, z is assumed as half of film thickness (~160 nm), a is the contact radius estimated by the projective contact area as measured in SEM images of indents (see Supplementary Fig. 7(a) and 7(b)), and P is the maximum load (500 μN). Though the Hertzian model was

oversimplified because of the non-spherical geometry of indenter as well as the remarkable plastic deformation and pile-up effects in NC Al films, Supplementary Fig. 7(c) unambiguously shows higher stresses close to indents, while the intensity of stress field was comparable in the two films (the more impure and mechanically stable film even underwent higher stress). Thus, this indicates a higher critical stress required for stress-driven microstructural evolution with the increase of C_O.

Supplementary Note 3 | Alignment process for in situ indentation tests

For each test, the cube-corner indenter of Hysitron PicoIndenter [14] was firstly driven in Z direction (i.e., forward) and X direction (i.e., parallel with the wedge) to approach an interested grain in the NC Al film on Si wedge [15], then in Y direction (i.e. parallel with incident electron beam) so that the tip end and the side surface of film were concurrently in focus, as shown in Fig. 2(b) of main text. The above coarse adjustments were performed mechanically. Subsequently, fine adjustments were performed using piezotube so that the tip end was well aligned with the film on the apex of wedge. Cross-sectional layout of the specimen is shown in Supplementary Fig. 8(a). First, the tip end was moved in X direction to the vicinity (typically ~ 100 nm) of the interested grain, and in Z direction to be close enough (typically ~ 10 nm, as viewed in projective image) to the film surface. Then, the indenter was moved in Y direction step by step (step length ~ 100 nm). For each step, a very shallow indentation with a fixed depth was attempted and the end load was recorded. The Y position yielding the maximum end load (see Supplementary Fig. 8(b)) was thus considered as the proper alignment condition to perform real tests on the nearby grain of interest.

Supplementary Note 4 | FEM of stress field distribution

In order to examine the applicability of Hertzian model to our *in situ* indentation tests, the stress field distribution near the indenter was calculated using FEM in ABAQUS 6.10

software [16]. As shown in Supplementary Fig. 9, the homogeneous half-space of bulk Al (i.e., the Hertzian model) was simulated using a rectangular solid with 1000 nm width along X and Y directions and 3150 nm height along Z direction. The geometry of Al film on Si wedge substrate was simulated based on the cross-sectional TEM image (see Supplementary Fig. 8(a)). The top 150 nm along Z direction of the half-space was kept as Al film, while the underlying 3000 nm was replaced with Si substrate. Then, the apex of wedge was simplified as a plateau with 150 nm width along Y direction, followed by 30° inclination on both sides until 1000 nm width. The width along X direction was kept as 1000 nm. All simulations were performed with mirror symmetry at both X = 0 and Y = 0, and only a quarter of each system was calculated and displayed hereinafter. Totally 54,000 linear tetragonal elements with 8-node were used in each simulation, with finer meshes used in Al film (or the top 150 nm of half-space) to help improve the precision. Nodes on the bottom surface of Si substrate (at Z = 3150 nm, origin of coordinates was set at the top surface of Al film before indentation) was fixed for translation in Z directions, and all other nodes were free to move in three directions. Both Al film and Si substrate were assumed to be elastic and isotropic, as supported by the low anisotropy of Al [17] and the insensitive dependence of our interested critical shear stress to reduced modulus (see details later). All simulations used elastic modulus (E) and Poisson's ratio (ν) of E_{Al} = 73 GPa, ν_{Al} = 0.35, E_{Si} = 150 GPa, ν_{Si} = 0.17 [18], and the indenter was simplified as a semi-spherical rigid body, since the diamond tip (E_{i} = 1171 GPa, ν_{i} = 0.07) was much stiffer than Al film and Si substrate. Indentation tests were simulated according to a representative experimental test on NC Al film with C_{O} of 0.7 at. % (see Fig. 3(i) and 5(a) of main text). Tip radius was 132.5 nm, and the maximum force in all simulations was set as the experimental critical force of 13.7 μN, corresponding to the onset of stress-driven grain growth. The friction coefficient was assumed to be zero.

Supplementary Fig. 9 also shows the distribution of shear stress in Al film (or the top 150 nm of half-space) at the maximum force. Qualitatively, the wedge-shaped Si

substrate induced no detectable difference in the shape of stress field in Al film. For more quantitative details, Supplementary Fig. 10 shows the shear stress plotted as functions of x and z across the maximum stress, and the simulated results were compared with the analytical solution of Hertzian model. Clearly, the influence of Si substrate was negligible, seeing that the stress field was rapidly decayed within Al film before penetration to Si substrate. Hence, the Hertzian model was confirmed to be a good approximation for our experimental configuration.

Supplementary Note 5 | Error analysis of critical shear stress

According to above FEM simulations, the stress fields in NC Al films on a Si wedge can be well approximated using the Hertzian model of elastic contact. Therefore, the following error analysis of critical shear stress (τ_{CR}) for GB motion was performed based on the Hertzian model. The two experimental tests presented in main text (see Fig. 3) were representatively studied herein.

Supplementary Fig. 11(a) shows the variation of τ_{CR} with the measurement error in critical force (ΔP). With a standard deviation in force, typically ± 0.2 μN (corresponding to a peak-to-peak noise level of ~1 μN), the variation in τ_{CR} was only on the order of ± 1%.

Supplementary Fig. 11(b) shows the variation of τ_{CR} with the value of reduced modulus, as determined by $E_r = (\frac{1-\nu_i^2}{E_i} + \frac{1-\nu_f^2}{E_f})^{-1}$. Assuming the elasticity of indenter (i) as that of diamond and the film (f) as bulk Al yielded an E_r = 77.6 GPa. However, seeing that the indenter was always adhered with substantial NC Al debris (see Fig. 3(a-h) of main text) lost from the films during indentation tests and the preceding alignment process, it was reasonable to assume similar elastic properties of the indenter and the film, which yielded an E_r = 41.6 GPa. Still, E_r could be lowered seeing that the modulus of NC Al films might be significantly lower than that of bulk Al [1,19], and a typical film

modulus of ~40 GPa yielded an E_r = 22.8 GPa. The plastic deformation preceding the activation of GB motion, as accommodated by discrete dislocation slips across the Al grains [20], also lead to the relaxation of stress, which could be simplified as a decrease of film stiffness (or E_r) assuming that there was no microstructural changes inside grains following the emission of a full dislocation [21] from GB source and its absorption in the opposing GB sink [20]. The measurement error in curvature radius of indenter (R) can be also included in the effect of E_r, since τ_{CR} was influenced by R only through the contact radius $a = (\frac{3PR}{4E_r})^{\frac{1}{3}}$, though the uncertainty of R was much smaller than that of E_r.

Overall, Supplementary Fig. 11(b) shows that τ_{CR} increased with E_r, however, the variation of τ_{CR} was only ± 3~9 %, as E_r varied between 22.8 GPa and 77.6 GPa. All data shown in main text were calculated using an E_r = 41.6 GPa.

We noticed that the most dominant uncertainty in τ_{CR} comes from the coordinate (r, z) of "hotspot" where the GB motion was initially observed. As representatively shown in Fig. 5(a) and 5(b) of main text, the measurement error of the position of "hotspot" along GB generated variation of τ_{CR} on the order of ± 10 %. Moreover, the position r = 0 was determined by fitting the circle of curvature of the tip, and the position z = 0 was determined by fitting the top surface of the film, both having measurement errors of about ± 5 nm. This generated an additional variation of τ_{CR} ranging in ± 10~20 % (dependent on test), as shown in Supplementary Fig. 12.

Taken as a whole, the uncertainty in critical shear stress for GB motion was determined by combining the contributions from force ($\Delta\tau_P$), reduced modulus and tip radius ($\Delta\tau_E$), coordinate of "hotspot" ($\Delta\tau_{rz}$), and position of coordinate origin ($\Delta\tau_0$), as follows:

$$\Delta\tau_{CR} = \sqrt{(\Delta\tau_P)^2 + (\Delta\tau_E)^2 + (\Delta\tau_{rz})^2 + (\Delta\tau_0)^2}\,. \qquad (2)$$

This ultimately generated $\Delta\tau_{CR}$ ranging in ± 12~23 % (dependent on test), as shown in Fig. 6(f) of main text. On the other hand, the measurement error in shear stress for the propagation of dislocations along GBs ($\tau_{CR} = Gb/L$) was simply estimated as:

$$\Delta\tau_{\mathrm{CR}} = \tau_{\mathrm{CR}} \cdot \frac{\Delta L}{L}, \qquad (3)$$

where G is shear modulus, b is Burgers vector, both of bulk Al, and L is the minimum diameter of the bowing dislocation segment observed before de-pinned from GB pinning points. ΔL was assumed as the width of dislocation line in dark field TEM images, on the order of 5 nm. The uncertainty of τ_{CR} was thus about ± 15 %, as shown in Fig. 6(f) of main text.

Supplementary References

[1] He, M. R. *et al.* Understanding the mechanical behavior of nanocrystalline Al-O thin films with complex microstructures. *Acta Mater.* **77**, 269–283 (2014).

[2] Sigle, W., Richter, G., Ruehle, M. & Schmidt, S. Insight into the atomic-scale mechanism of liquid metal embrittlement. *Appl. Phys. Lett.* **89**, 121911 (2006).

[3] Felfer, P., Ceguerra, A. V., Ringer, S. P. & Cairney, J. M. Detecting and extracting clusters in atom probe data: A simple, automated method using Voronoi cells. *Ultramicroscopy* **150**, 30–36 (2015).

[4] Krakauer, B. W. & Seidman, D. N. Absolute atomic-scale measurements of the Gibbsian interfacial excess of solute at internal interfaces. *Phys. Rev. B* **48**, 6724–6727 (1993).

[5] Herbig, M. *et al.* Atomic-Scale Quantification of Grain Boundary Segregation in Nanocrystalline Material. *Phys. Rev. Lett.* **112**, 126103 (2014).

[6] Araullo-Peters, V. J. *et al.* Atom probe crystallography: Atomic-scale 3-D orientation mapping. *Scripta Mater.* **66**, 907–910 (2012).

[7] Yao, L., Ringer, S. P., Cairney, J. M. & Miller, M. K. The anatomy of grain boundaries: Their structure and atomic-level solute distribution. *Scripta Mater.* **69**, 622–625 (2013).

[8] Trimby, P. W. Orientation mapping of nanostructured materials using transmission Kikuchi diffraction in the scanning electron microscope. *Ultramicroscopy* **120**, 16–24 (2012).

[9] Trimby, P. W. *et al.* Characterizing deformed ultrafine-grained and nanocrystalline materials using transmission Kikuchi diffraction in a scanning electron microscope. *Acta Mater.* **62**, 69–80 (2014).

[10] Merkle, K. L. & Wolf, D. Low-energy configurations of symmetric and asymmetric tilt grain boundaries. *Phil. Mag. A* **65**, 513–530 (1992).

[11] Tschopp, M. A. & McDowell, D. L. Structural unit and faceting description of

Σ3 asymmetric tilt grain boundaries. *J. Mater. Sci.* **42**, 7806–7811 (2007).

[12] Brandon, D. G. The structure of high-angle grain boundaries. *Acta Metall.* **14**, 1479–1484 (1966).

[13] Feng, G., Qu, S., Huang, Y. & Nix, W. D. An analytical expression for the stress field around an elastoplastic indentation/contact. *Acta Mater.* **55**, 2929–2938 (2007).

[14] Minor, A. M. *et al.* A new view of the onset of plasticity during the nanoindentation of aluminum. *Nat. Mater.* **5**, 697–702 (2006).

[15] Jin, M., Minor, A. M., Stach, E. A. & Morris, J. W. Direct observation of deformation-induced grain growth during the nanoindentation of ultrafine-grained Al at room temperature. *Acta Mater.* **52**, 5381–5387 (2004).

[16] ABAQUS 6.10. Dassault Systèmes Simulia Corp., Providence, RI.

[17] Gianola, D. S. *et al.* Stress-assisted discontinuous grain growth and its effect on the deformation behavior of nanocrystalline aluminum thin films. *Acta Mater.* **54**, 2253–2263 (2006).

[18] Saha R. & Nix, W. D. Effects of the substrate on the determination of thin film mechanical properties by nanoindentation. *Acta Mater.* **50**, 23–38 (2002).

[19] Haque, M. A. & Saif, M. T. A. Deformation mechanisms in free-standing nanoscale thin films: A quantitative in situ transmission electron microscope study. *Proc. Natl. Acad. Sci. USA* **101**, 6335–6340 (2004).

[20] Yamakov, V., Wolf, D., Phillpot, S. R., Mukherjee, A. K. & Gleiter, H. Dislocation processes in the deformation of nanocrystalline aluminium by molecular-dynamics simulation. *Nat. Mater.* **1**, 45–48 (2002).

[21] Van Swygenhoven, H., Derlet, P. M. & Frøseth, A. G. Stacking fault energies and slip in nanocrystalline metals. *Nat. Mater.* **3**, 399–403 (2002).
